# A clinical trial protocol to treat massive Africanized honeybee (*Apis mellifera*) attack with a new apilic antivenom

**DOI:** 10.1186/s40409-017-0106-y

**Published:** 2017-03-16

**Authors:** Alexandre Naime Barbosa, Leslie Boyer, Jean-Philippe Chippaux, Natalia Bronzatto Medolago, Carlos Antonio Caramori, Ariane Gomes Paixão, João Paulo Vasconcelos Poli, Mônica Bannwart Mendes, Lucilene Delazari dos Santos, Rui Seabra Ferreira, Benedito Barraviera

**Affiliations:** 1 Department of Tropical Diseases, Botucatu Medical School, São Paulo State University (UNESP – Univ Estadual Paulista), Botucatu, SP Brazil; 2 VIPER Institute, University of Arizona College of Medicine, Tucson, AZ USA; 3 CERPAGE, Faculté des Sciences de la Santé, Université d’Abomey-Calavi, Cotonou, Benin; 4 UMR216, Mère et enfant face aux infections tropicales and PRES Sorbonne Paris Cité, Faculté de Pharmacie, Université Paris Descartes, Paris, France; 5 Clinical Research Unit (UPECLIN), Botucatu Medical School, São Paulo State University (UNESP – Univ Estadual Paulista), Botucatu, SP Brazil; 6 Department of Internal Medicine, Botucatu Medical School, São Paulo State University (UNESP - Univ Estadual Paulista), Botucatu, SP Brazil; 7 Center for the Study of Venoms and Venomous Animals, São Paulo State University (UNESP – Univ Estadual Paulista), Av. José Barbosa de Barros, 1780, Fazenda Experimental Lageado, Botucatu, SP CEP 18.610-307 Brazil

**Keywords:** *Apis mellifera*, Bee venom, Toxins, Envenomation, Heterologous serum, Apilic antivenom, Bee antivenom

## Abstract

**Background:**

Envenomation caused by multiple stings from Africanized honeybees *Apis mellifera* constitutes a public health problem in the Americas. In 2015, the Brazilian Ministry of Health reported 13,597 accidents (incidence of seven cases per 100,000 inhabitants) with 39 deaths (lethality of 0.25%). The toxins present in the venom, which include melittin and phospholipase A_2_, cause lesions in diverse organs and systems that may be fatal. As there has been no specific treatment to date, management has been symptomatic and supportive only.

**Methods:**

In order to evaluate the safety and neutralizing capacity of a new apilic antivenom, as well as to confirm its lowest effective dose, a clinical protocol was developed to be applied in a multicenter, non-randomized and open phase I/II clinical trial. Twenty participants with more than five stings, aged more than 18 years, of both sexes, who have not previously received the heterologous serum against bee stings, will be included for 24 months. The proposed dose was based on the antivenom neutralizing capacity and the number of stings. Treatment will be administered only in a hospital environment and the participants will be evaluated for a period up to 30 days after discharge for clinical and laboratory follow-up.

**Results:**

This protocol, approved by the Brazilian regulatory agencies for ethics (National Commission for Ethics on Research – CONEP) and sanitation (National Health Surveillance Agency – ANVISA), is a guideline constituted by specific, adjuvant, symptomatic and complementary treatments, in addition to basic orientations for conducting a clinical trial involving heterologous sera.

**Conclusions:**

This is the first clinical trial protocol designed specifically to evaluate the preliminary efficacy and safety of a new antivenom against stings from the Africanized honeybee *Apis mellifera*. The results will support future studies to confirm a new treatment for massive bee attack that has a large impact on public health in the Americas.

## Background

African *Apis mellifera scutellata* bees were introduced into the southeastern region of Brazil in 1956. Twenty-six queens swarmed and initiated the Africanization of the American continent. These new hybrids, known as Africanized honeybees, are very defensive and attack *en masse*, causing serious injuries in humans and other animals. These bees have expanded their range and today are found from Argentina to the United States of America [1, 2]. Consequently, public health authorities in Brazil included bee sting events as an object of epidemiological vigilance as a consequence of the growing number of victims and of deaths related to this envenomation.

A recent epidemiological study made it possible to specify the factors concerning the incidence and severity of bee stings in Brazil [3]. They represented 6% of cases of envenomation and 9% of deaths due to animal envenomation, which denotes a high severity of bee stings. The incidence predominated in southern Brazil. Stings frequently occurred during recreational and professional activities, both in suburban and rural settings, and this explained why the incidence was fairly distributed for all ages, although the masculine gender was more involved. However, two thirds of the patients stung by bees arrived at the hospital less than three hours after the bee stings, which is very different from all other envenomated patients who arrived significantly later. Asymptomatic and mild envenomation accounted for up to 90% of cases, moderate envenomation for 10–18% and severe ones for 0.8–1.3%. The case fatality rate was 0.3 to 0.4% in all regions. Nevertheless, the study does not specify whether it was single or multiple punctures, i.e. it cannot be determined whether death is related to anaphylactic shock or envenomation after inoculation with a large amount of bee venom.

**Fig. 1:**
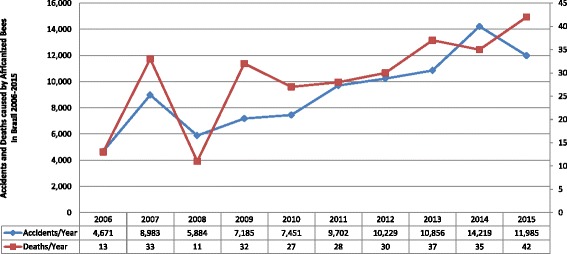
Temporal distribution of the number of cases reported for Africanized honeybee sting in Brazil between 2000 and 2015 [4]

In 2000, 1,440 cases, including three deaths, were reported; in 2015 this number grew to 13,597 cases with 39 deaths, that is, an increase of almost tenfold in the number of cases in 15 years [3, 4]. Despite this impressive increase in reported incidence and mortality, many regional specialists believe that these numbers remain underreported. Fig. 1 below demonstrates this growth.

The physiopathology of this accident is complex due to the interaction between the diverse toxic components of the venom, the potential target organs and the immune response of the victim [5, 6]. Generally, bee stings can present two different outcomes:

- Anaphylaxis: occurs in individuals allergic to bee venom. In these cases a single sting can cause serious generalized allergic reactions, possibly leading to death. These consequences are not related to the toxicity of the venom due to the small quantity inoculated. Treatment is limited to general measures and the use of symptomatic drugs aiming to control the anaphylaxis [7].
- Direct toxicity from the venom: occurs when there are a substantial number of stings – generally more than 200 in adults. In this case, the venom volume is sufficient to cause damage to vital organs. Prominent among the toxic components are melittin and phospholipase A_2_, whose presence accounts for more than 60% of the dry weight of the venom. Direct injury to the cardiovascular, muscular, neurological, dermatological, metabolic, hematological, respiratory and renal systems can lead to death [7, 8].

The clinical manifestations resulting from multiple stings are: generalized pain, intense pruritus, flushing, hyperthermia, papules, urticarial plaques, hypotension, tachycardia, headache, nausea, vomiting, abdominal colic, bronchospasms and psychomotor agitation, which may subsequently evolve to a state of torpor, accompanied by respiratory and cardiac failure and, principally, severe renal failure [8–10]. The most common laboratory alterations are: leukocytosis with neutrophilia, hemolysis, thrombocytopenia, disseminated intravascular coagulation, rhabdomyolysis with increase of creatine phosphokinase (CPK), elevation of the enzymes aspartate aminotransferase (AST) and alanine amino transferase (ALT), increase of urea, creatinine and myoglobin in the urine [8–10].

Mortality is elevated at the extremes of age [4]. In children, due to lower body mass, envenomation can be much more severe, since with many stings the concentration of the toxic components is elevated. In the elderly, greater risk involves such preexisting comorbidities as chronic renal, cardiac or respiratory failure. These constitute an important additional risk factor in patients who are victims of multiple stings.

Given that until the present there has been no specific treatment, a consortium of Brazilian researchers developed the first antivenom for bee stings constituted of heterologous antibodies against the toxins from the venom and approved for clinical trial. In order to bridge the gap between the laboratory workbench and the patient, the objectives of this study were to standardize a clinical protocol applicable to patients affected by multiple stings from Africanized *Apis mellifera* honeybees, to evaluate safety and preliminary efficacy, and to standardize the lowest dose of the new antivenom. The absence of a previous standardized clinical protocol justifies its publication in order to open a discussion on the relevance of antivenom strategy and methodology proposed.

## Methods

### Antivenom development and dose proposal

Researchers from the Center for Studies of Venoms and Venomous Animals (CEVAP) of the São Paulo State University (UNESP) in partnership with the Vital Brazil Institute (IVB), Brazil, developed the apilic antivenom. For this, the principal toxins from the venom of Africanized honeybee maintained at the Lageado Experimental Farm, UNESP campus in Botucatu, São Paulo, Brazil, were extracted and purified. Next, previously selected horses were immunized with increasing doses of the chosen antigens. These protocols are described in detail in the developed guide for researchers and in the patent submitted [7, 11].

The proposed dose of the new antivenom was calculated taking into account the premises of the *WHO Guideline for the production control and regulation of snake antivenom immunoglobulins* [12] that prioritize the quantity of venom inoculated in the host, neutralization potency and the proposed antivenom dose. For these calculations, the authors relied on the fact that one bee inoculates during one sting approximately 0.1 mg of venom [1]. According to the serum neutralization tests, each milliliter of standardized antivenom neutralizes 1.25 mg of venom. Therefore, 10 mL of antivenom should neutralize the venom of 100 stings. The experimental validation of antivenom efficacy and preclinical tests are detailed in a paper in progress.

### Design of the *Apis* study

This is a multicenter, non-randomized and open phase I/II clinical trial study to evaluate the safety, determine the pharmacokinetic and proteomic profile, and confirm the lowest antivenom dose, according to the severity of each case. It will include 20 adult individuals, of both sexes, afflicted with multiple stings from Africanized *Apis mellifera* honeybees. The study population will be of the type non-probabilistic by convenience (or accidental) sample, given the low demographic density of the studied phenomenon and the extreme geographic decentralization of this event. The sample size was estimated by considering examples of phase I/II clinical studies proposing to study the safety and neutralizing power and to confirm the minimum required dose of antivenom [12]. Not performing the sample calculation is justified by the fact that the efficacy outcome is not the target at this moment given that there are no data on safety and adequate dose. The study will last 36 months, with 24 months for recruitment.

### Objectives

- Primary objectives: to evaluate the safety of the antivenom including number and severity of acute adverse events, as well as deaths suspected to be related to the intervention; and to confirm the lowest effective dose when faced with different quantities of venom inoculated in patients exposed to multiple stings from Africanized honeybees.
- Secondary objectives: to correlate the severity of the initial clinical condition with number of stings.
- Explanatory objectives: to evaluate the antivenom neutralizing power by pharmacokinetic and proteomic studies; and to evaluate the antivenom pharmacokinetics and immunogenicity.

### Outcomes

- Primary outcomes: to evaluate the antivenom safety profile through laboratory and clinical adverse events; to verify the proportion of individuals with improvement in the initial clinical picture by monitoring signs, symptoms and laboratory exams.
- Secondary outcomes: to evaluate the degree de correlation between the number of stings and the severity of the initial clinical picture (APACHE II).
- Exploratory outcomes: to evaluate the pharmacokinetic and immunogenic profile of antivenom/venom at doses established by an analysis of the blood samples collected at different moments (before and at 2, 6, 12 and 24 h after heterologous serum therapy); to evaluate the proteomic profile of the antivenom as determined by the differential expression of the proteins albumin, C-reactive protein (CRP) and fibrinogen, by the presence of the components melittin and phospholipase A_2_, and by the heterologous serum protein profile of the degradome and peptide/protein biomarkers.

### Study participation agreement

Prior to the performance of any procedure related to the study, the participants in the research and/or their family members must be informed about its objectives, risks and procedures. Those who consented to participate must sign, date and initial the terms of free and informed consent (TFIC). The process of obtaining the TFIC must be documented on the patient’s chart. If the consent and signature of the patient are impossible to obtain, by virtue of his or her clinical condition, it is essential to locate a family member or other responsible party to provide them.

### Approval from regulatory agencies

#### Approval from the Research Ethics Committee (CEP)

The National Commission for Ethics on Research (CONEP) approved the clinical research protocol and the terms of free and informed consent (TFIC). The investigators of the other centers must send to the coordinating center a copy of the approval by the local research ethics committee (CEP) of the current version of the clinical study protocol, the TFIC and its necessary revisions. The coordinating CEP-CONEP system will oversee the conduct of the study.

#### Approval from the management of ANVISA Clinical Research

The National Health Surveillance Agency (ANVISA), through intermediation of the General Management of Drugs department, received all documentation of the product, including the clinical research protocol, the guide for researchers (a complete history of the development of the product) and the other documents necessary for analysis and approval. This is the first time that an antivenom against bee stings is evaluated in human’s clinical trial.

#### Registration in the ReBec

The Brazilian Clinical Trials Registry (ReBec) is a virtual platform of free access to a registry of experimental and non-experimental studies performed on humans and conducted on Brazilian territory, by Brazilian and foreign researchers. ReBec is a joint project of the Ministry of Health (DECIT/MS), the Pan American Health Organization (PAHO) and Oswaldo Cruz Foundation (FIOCRUZ). The executive committee of ReBec is composed of the abovementioned institutions and ANVISA. The *Apis* study was registered in ReBec http://www.ensaiosclinicos.gov.br/.

### Centers participating in the study

All the Brazilian research centers registered in the National Clinical Research Network (RNPC) will be invited to participate.

### Statistical analysis

The statistical analysis, as well as the choice of tests for comparison among the research participants, will be executed with respect to the presuppositions determined by the results, characteristics and behavior of the variables in the study. The binomial variables will be compared by chi-square and Fisher’s exact test. The numerical variables will be compared by the Student’s *t* test or *U* test of Mann–Whitney [13, 14].

## Results and Discussion

### Regulatory agencies and participating centers

The clinical trial was registered in ReBec on August 21, 2014; approved by the Research Ethics Committee at the Botucatu Medical School (CAAE: 19006813.4.1001.5411) on October 21, 2013 (clinical protocol version 1), on December 1, 2014 (clinical protocol version 2) and on June 6, 2016 (clinical protocol version 3). The study was authorized by ANVISA on February 5, 2016 (Special Communication n. 11/2016, Process n. 25351.611582/2014-93, CE Expedient: 1215967161).

Three research centers registered in the National Clinical Research Network (RNPC) applied for and fulfilled the regulatory prerequisites, namely:

- Clinical Research Unit (UPECLIN) of the Botucatu Medical School, UNESP, this is the coordinating center.
- Clinical Research Center at Hospital Nossa Senhora da Conceição, at the University of South Santa Catarina (UNISUL) located in Tubarão, Santa Catarina state.
- University Hospital at the Medical School of Federal University of Triângulo Mineiro (UFTM) located in Uberaba, Minas Gerais state.

### Standardization of criteria for inclusion, exclusion and discontinuation

Participants shall meet the following criteria.

### Inclusion

- Having an age above 18 years for both sexes.
- Having diagnosis of an accident with bees of the genus *Apis*.
- Having agreement from the patient or a responsible family member to receive the antivenom.

### Exclusion

- Having had a previous adverse reaction to heterologous serum produced in horses.
- Being pregnant.
- Having a chronic disease, including congenital or acquired immunodeficiency.

### Discontinuation

- Developing anaphylactic shock resistant to the management protocol for reactions of acute hypersensitivities.
- Withdrawing from the terms of free and informed consent.

### Plan of treatment (specific, adjuvant, symptomatic and complementary)

#### Specific treatment

The *WHO Guideline for the production control and regulation of snake antivenom immunoglobulins* [12] suggests that the protocol prepared for the specific treatment be based on the estimated quantity of venom inoculated by the bee and on the neutralizing capacity of the antivenin. Thus, the authors suggested:

- Up to 5 stings: specific treatment is not indicated, except for a medical criterion.
- Between 5 and 200 stings: two vials of apilic antivenom.
- Between 201 and 600 stings: six vials of apilic antivenom.
- Above 600 stings: ten vials of apilic antivenom.

After the dose is determined by the attending medical team, the vials must be opened and their content aspirated by means of a syringe. Next, insert all the content into a flask containing 250 mL of normal 0.9% saline solution, previously emptied. This flask containing the antivenom must be connected into a Y formation with another flask containing 250 mL of 0.9% saline. One large-caliber vein of the forearm must be catheterized and these two contents need to be infused in the Y within two hours. All patients must be hospitalized and monitored throughout the infusion by the continuous presence of the hospital staff at the patient’s bedside. The team must also have at their disposal the drugs and equipment necessary for the treatment of mild or severe adverse reactions. Any adverse events and signs and symptoms of possible toxicity must be noted on the patient’s chart.

#### Adjuvant treatment

The adjuvant treatment aims to maintain the patient and avoid hemodynamic shock, preserve kidney function, diminish cerebral edema and prevent the dysfunctions resulting from hemoglobinuria. Thus, the following is proposed:

- Replenish volume by hydrating patient vigorously with 0.9% saline, after catheterization of a large-caliber peripheral vein, in order to ensure hemodynamic stability. Always maintain arterial pressure levels above 90 × 60 mmHg.
- Use vasoactive drugs including dopamine and/or noradrenaline to treat hypotension refractory to the volume, at the discretion of the medical staff.
- Suspect rhabdomyolysis when the level of creatine phosphokinase (CPK) is above 5,000 U/mL. The presence of dark urine, of oliguria and/or anuria may also denote the presence of rhabdomyolysis. In this case the volume of 0.9% saline to be infused will be 20 mL/kg, running freely, being repeatable up to three times. The objective will be to maintain a urinary volume between 2 and 3 mL/kg/hour. Vigorous hydration must be maintained until CPK reaches levels below 1,000 U/mL.
- In the presence of refractory anuria or oliguria, require an evaluation from a nephrologist for potential hemodialysis.
- In the presence of electrolytic disturbances such as alterations in levels of Na^+^, K^+^, Ca^++^ or Mg^++^, these parameters should be closely monitored. Hyperkalemia and hypocalcemia, when present, must be corrected according to the protocols of the referring services.

#### Symptomatic treatment

At the discretion of the medical team, goals for the patient include:

- To treat and prevent hypersensitivity reactions inherent to venom or antivenom. For this, use:
  - ➢ antihistamines – inject intramuscularly, one vial of 50 mg of promethazine or similar option upon the arrival of the patient; repeat every six hours if necessary;
  - ➢ corticosteroids – administer intravenously, 200 mg of hydrocortisone or similar option upon the patient’s arrival; repeat every six hours if necessary. This scheme could be maintained for 3 to 5 days, according to the clinical evolution;
  - ➢ when anaphylactic shock is suspected – if the patient presents severe hypotension and in the absence of a palpable pulse, inject subcutaneously 500 μg (0.5 mL) of aqueous adrenaline 1:1,000.
- Treat the pain: inject intramuscularly, one vial of petidine chlorhydrate 50 mg or similar; repeat every six hours if necessary.
- In the presence of bronchospasm: utilize a catheter of oxygen (O_2_) associated with bronchodilators of the type β-2-agonist inhalants (salbutamol, phenoterol or terbutaline), at customary doses used at the referring center. Continue as needed until the disappearance of symptoms.

#### Complementary treatment

- Catheterize a large-caliber peripheral vein. In critical patients use central venous access.
- Apply cardiac monitor and O_2_ saturation meter.
- Removal of the stingers should be performed immediately after stabilization of the clinical parameters of the patient. The count will help estimate the amount of antivenom to be administered.

#### Other observations

- Apply vesical and nasogastric probe when indicated.
- Apply potassium permanganate diluted 1:40.000, for antisepsis of the skin affected by the stings.
- Apply enteral feeding containing about 2,000 cal per day when indicated.
- Maintain fluids, electrolytes and acid–base status as indicated.
- Perform tracheotomy and/or oral-tracheal intubation with mechanical ventilation, when indicated.
- Perform peritoneal dialysis and/or hemodialysis, when there is acute kidney failure.
- Prevent the formation of bedsores.
- Avoid secondary respiratory infections.

### Conduct according to the number of stings and the presence or absence of anaphylactic reaction

#### Up to five stings

- Without anaphylactic shock:
  - ➢ remove all of the stingers correctly;
  - ➢ prescribe hydrocortisone ointment in isolation or associated with 5% menthol;
  - ➢ prescribe dextrochlorpheniramine 6 mg orally, every eight hours as needed;
  - ➢ apilic antivenom is not indicated.
- In the presence of anaphylactic shock:
  - ➢ catheterize access of a large-caliber or central vein to hydrate the patient;
  - ➢ follow the protocol contained in the symptomatic treatment item;
  - ➢ remove all the stingers correctly;
  - ➢ apilic antivenom is not indicated.

#### Up to 200 stings

- Without anaphylactic shock:
  - ➢ remove all the stingers correctly;
  - ➢ catheterize a central vein for hydration of the patient;
  - ➢ follow the protocol contained in the symptomatic treatment item;
  - ➢ always hospitalize the patient;
  - ➢ apply two vials of apilic antivenom.
- In the presence of anaphylactic shock:
  - ➢ catheterize a central vein for hydration of the patient;
  - ➢ follow the protocol contained in the symptomatic treatment item;
  - ➢ remove all the stingers correctly;
  - ➢ these individuals must always be hospitalized and may require treatment in an intensive care unit (ICU);
  - ➢ apply two vials of apilic antivenom.

#### Up to 600 stings

- Without anaphylactic shock:
  - ➢ catheterize a central vein for hydration of the patient;
  - ➢ follow the protocol contained in the symptomatic treatment item;
  - ➢ remove all the stingers correctly;
  - ➢ these individuals must always be hospitalized in an ICU;
  - ➢ apply six vials of apilic antivenom.
- In the presence of anaphylactic shock:
  - ➢ catheterize a central vein for hydration of the patient;
  - ➢ follow the protocol contained in the symptomatic treatment item;
  - ➢ remove all the stingers correctly;
  - ➢ these individuals may need to be hospitalized in an ICU;
  - ➢ apply six vials of apilic antivenom.

#### More than 600 stings

- Without anaphylactic shock:
  - ➢ catheterize a central vein for hydration of the patient;
  - ➢ follow the protocol contained in the symptomatic treatment item;
  - ➢ remove all the stingers correctly;
  - ➢ these sick individuals in general require hospitalization in an ICU;
  - ➢ apply ten vials of apilic antivenom.
- In the presence of anaphylactic shock:
  - ➢ catheterize a central vein for hydration of the patient;
  - ➢ follow the protocol contained in the symptomatic treatment item;
  - ➢ remove all the stingers correctly;
  - ➢ these sick individuals in general require hospitalization in an ICU;
  - ➢ apply ten vials of apilic antivenom.

### Clinical parameters

Product safety will also be evaluated through the clinical parameters verified in the evaluations, via both laboratory exams and adverse events that will have occurred during the study.

#### Adverse event (AE)

According to the International Council for Harmonisation of Technical Requirements for Pharmaceuticals for Human Use (ICH) [15], an adverse event is any undesirable clinical occurrence in a patient or participant in clinical research that receives or utilizes a pharmaceutical product and that does not necessarily present a causal relation to this treatment. Thus, an adverse event can be any unfavorable unintentional signal (including an abnormal laboratory finding, symptom or disease temporally associated with the use of a product under investigation, considered related or not to it). Preexisting conditions that worsen during a study must be reported as an AE. The events observed during a clinical study should be reported in the patient’s chart (source document), on the page of events of the electronic clinical registry and must be classified as to:

- Intensity
  - ➢ minimal (degree I): discomfort perceived, but without interruption of normal daily activity;
  - ➢ moderate (degree II): discomfort sufficient to reduce or inhibit daily activity;
  - ➢ severe (degree III): incapacity to work or perform normal daily activity;
  - ➢ risk to life (degree IV): represents an immediate risk to life;
  - ➢ death (degree V).
- Causal relation
  - ➢ **probable**: the temporal relation is well defined without the existence of another possible causal factor. In this case, there is an almost certain relationship between the reaction and the medicament;
  - ➢ **possible**: a temporal relation between an event and the administration of a medicament is well defined, but there is another possible causal factor;
  - ➢ **remote**: a relationship with the medicament is improbable, but cannot be definitively discarded;
  - **➢ not related**: a temporal relationship between an event and the ingestion or administration of a medicament is nonexistent or doubtful, or there exists another factor that may be identified as a causal factor of the reaction.

##### Observation

For the current study, an adverse event is considered any unfavorable and unintended signal that occurs after administration of the antivenom against bee stings. Conditions prior to such administrations must be registered as medical history.

#### Serious adverse event (SAE)

A serious adverse event is an AE that occurs during any phase of the study and that meets the following criteria: it threatens life; it results in death or a significant or permanent incapacity, congenital anomaly, hospitalization or the prolongation of an existent condition.

Serious adverse events also should be classified according to the guidelines of the previous item, filled out in the SAE formulary, sent to the coordinating center within 24 h of obtaining the knowledge by means of a virtual study platform and should be reported concomitantly with the system CEP-CONEP. The coordinating center will immediately notify ANVISA and subsequently the Department of Science and Technology in the Ministry of Health (DECIT/SCTIE/MS).

#### Pregnancy

The occurrence of pregnancy during the study will need to be communicated immediately to the coordinating center via a pregnancy report formulary and reported concomitantly to the system CEP-CONEP. For such an occurrence, the participant would need to discontinue participation in the study.

#### Risks and benefits

- Risks:
  - Acute hypersensitivity to heterologous serum.
  - Adverse events intrinsic to heterologous serum.
  - Delayed hypersensitivity to heterologous serum.
- Benefits:
  - Neutralization of inoculated venom, with interruption of its toxicity.
  - Support to anaphylactic reactions related to envenomation.

### Subsidiary laboratory tests

To evaluate the safety parameters, tests will be required on hospitalization days one, two, five and ten, and at follow up (10, 20 and 30 days after hospital discharge) (Table 1).

**Table 1:** Laboratory tests requested during hospitalization and follow-up

Procedures	HOSPITALIZATION			FOLLOW-UP		
	Day 1	Day 2	Hospital discharge	Return 1 (R1) (10 days after discharge)	Return 2 (R2) (20 days after discharge)	Return 3 (R3) (30 days after discharge)
Application of FICT	X					
Demographic data	X					
Clinical history	X					
Clinical exam	X	X	X	X	X	X
Concomitant medications	X	X	X	X	X	X
Adverse events	X	X	X	X	X	X
Vital signs	X^a^	X	X	X	X	X
Hemogram	X	X	X	X	X	X
Urine type I	X^b^	X	X	X	X	X
Urea	X	X	X	X	X	X
Creatinine	X	X	X	X	X	X
AST	X	X	X	X	X	X
ALT	X	X	X	X	X	X
Alkaline phosphatase	X	X	X	X	X	X
Gama GT	X	X	X	X	X	X
Bilirubin (direct)	X	X	X	X	X	X
Bilirubin (indirect)	X	X	X	X	X	X
Fasting blood sugar	X	X	X	X	X	X
Cholesterol (total and fractions)	X	X	X	X	X	X
Fibrinogen	X^b^	X	X	X	X	X
C-reactive protein	X^b^	X	X	X	X	X
ESR	X^b^	X	X	X	X	X
PT	X^b^	X	X	X	X	X
APTT	X^b^	X	X	X	X	X
CPK	X^b^	X	X	X	X	X
Albumin	X	X	X	X	X	X
Globulin	X	X	X	X	X	X
Pharmacokinetic profile	X^c^	X	X	X	X	X
Proteomic profile	X^d^	X	X	X	X	X
Pregnancy test	X^e^					

*FICT* free and informed consent term, *AST* aspartate aminotransferase, *ALT* alanine amino transferase, *gama GT*: gamma glutamyl transferase, *CRP* C-reactive protein, *ESR* erythrocyte sedimentation rate, *PT* prothrombin time, *APTT* activated partial thromboplastin time, *CPK* creatine phosphokinase

^a^The vital signs should be taken every 30 min during the infusion and every hour through the first 12 h after the infusion

^b^Collect before and 12 h after termination of the infusion

^c^Evaluate the profile and level of the antibodies IgG and IgM against the antivenom infusion (before and at 2, 6, 12 and 24 h after infusion)

^d^Evaluate the profile of the different heterologous serum proteins in the antivenom (before and at 2, 6, 12 and 24 h after infusion)

^e^In special cases and at the investigator’s discretion this test could be repeated

Complementing the results of the laboratory exams, a proteomic evaluation will also be performed by degradome analysis, seeking to evidence eventual biomarkers responsible for physiological and clinical alterations [16, 17]. Furthermore, a pharmacokinetic profile of venom and of a new antivenom will be performed utilizing immunoenzymatic methods (ELISA) due to their sensitivity, reproducibility, ease of execution and low cost. Thus, the trial denominated “venenomia” will have the objective of determining the kinetics of circulating toxins and antivenom, aiding in the diagnosis, in the determination of accident severity and in the evaluation of heterologous serum therapy efficacy [18–20].

### Management of documents and publication policy

According to the law, all data obtained should be treated with discretion to ensure the privacy rights of the participant. The coordinating center must review the source document (case reports, charts and medical registries) for confirmation and registration. The research ethics committee (CEP) of each partner institution must approve the clinical research protocol, the the guide for researchers, the terms of free and informed consent (TFIC) and other information for the recruitment of patients, in addition to accompanying the conduction of the study. All the documentation (source documents, e-CRFs, laboratory exams, registries of medication dispensations in the study, correspondence related to the CEP and other annotations), must be kept on file for at least five years, in a location of restricted access. The coordinating institution undertakes to publish and disseminate the results obtained in an indexed peer-reviewed journal with a high impact factor. The choice of authors and collaborators will be based on the number of patients included, on the quantity of selection failures and on adherence to the procedures proposed. Moreover, if in the future any participating investigator wishes to use data from this study in publications or presentations, he or she will have to communicate such intention to the coordinating center 60 days prior to submission.

## Conclusions

Envenomation from bee stings are of great epidemiological importance in Brazil due to the high annual numbers of cases and deaths. This scenario may be even worse because of incomplete reporting by the attending health facilities. As the Africanized bee *Apis mellifera* is not restricted to Brazilian territory, the number of victims throughout the American continent is still unknown. Despite the relevance of the envenomation a double gap is perceived in the attention to victims of multiple stings, namely: the lack of standardized protocol with measures in the care of basic and advanced supports to life; and the lack of a specific antidote that inactivates the toxic fractions of this venom.

This study intends to propose a protocol of measures that include a triad of treatments standardized for the stabilization and general support of the patient (adjuvant, symptomatic and complementary), in addition to a specific treatment with a new antivenom, whose safety and preliminary efficacy will be evaluated. An important measure of the complementary treatment, for example, is the fast removal of the stingers after a massive attack, which should be performed immediately after stabilization of the clinical parameters of the patient [21, 22]. The count will help to estimate the amount of antivenom to be administered.

The nonperformance of conventional phase I clinical trials in healthy individuals to evaluate novel antivenoms produced in animals is justified by the risk of immediate reactions (anaphylaxis) and late reactions (heterologous serum disease) resulting from potential future exposures. Given the existence in Brazil of 13 available heterologous sera produced in horses, it cannot be ruled out that eventually one participant in “voluntary” research for a phase I conventional study may in the future require treatment by one of these products. This is a general agreement among researchers and official documents that address the theme, especially the *WHO Guideline for the production control and regulation of snake antivenin immunoglobulins* [12, 23–25].

This initiative, previously unpublished in the global literature, will have the potential to generate results that corroborate subsequent phases of the study, with the objective of making viable a specific treatment for this important, but neglected risk to public health in the American continent. Furthermore, the *Apis* study is meant to bring together measures for the support and general stabilization of patients who are victims of multiple bee stings, an endeavor of fundamental usefulness for health professionals in Brazil and other countries affected by this problem.

## Abbreviations

AE: Adverse event
ALT: Alanine amino transferase
ANVISA: Brazilian National Health Surveillance Agency
AST: Aminotransferase
CEP: Research Ethics Committee
CEVAP: Center for Studies of Venoms and Venomous Animals
CONEP: National Commission for Ethics on Research
CPK: Creatine phosphokinase
CRP: C-reactive protein
FIOCRUZ: Oswaldo Cruz Foundation
ICU: Intensive care unit
IVB: Vital Brazil Institute
PAHO: Pan American Health Organization
ReBec: Brazilian Clinical Trials Registry
RNPC: National Clinical Research Network
SAE: Serious adverse event
TFIC: Term of free and informed consent
